# Stepwise Proliferation and Chondrogenic Differentiation of Mesenchymal Stem Cells in Collagen Sponges under Different Microenvironments

**DOI:** 10.3390/ijms23126406

**Published:** 2022-06-08

**Authors:** Jing Zheng, Yan Xie, Toru Yoshitomi, Naoki Kawazoe, Yingnan Yang, Guoping Chen

**Affiliations:** 1 Research Center for Functional Materials, National Institute for Materials Science, 1-1 Namiki, Tsukuba, Ibaraki 305-0044, Japan; mr.oldcat@gmail.com (J.Z.); xieyanmpe@gmail.com (Y.X.); yoshitomi.toru@nims.go.jp (T.Y.); kawazoe.naoki@nims.go.jp (N.K.); 2 Department of Materials Science and Engineering, Graduate School of Pure and Applied Sciences, University of Tsukuba, 1-1-1 Tennodai, Tsukuba, Ibaraki 305-8577, Japan; 3 Graduate School of Life and Environmental Science, University of Tsukuba, 1-1-1 Tennodai, Tsukuba, Ibaraki 305-8572, Japan; yo.innan.fu@u.tsukuba.ac.jp

**Keywords:** collagen sponge, hydrogel, chondrogenesis induction factor, mesenchymal stem cells, proliferation, chondrogenesis, cartilage tissue engineering

## Abstract

Biomimetic microenvironments are important for controlling stem cell functions. In this study, different microenvironmental conditions were investigated for the stepwise control of proliferation and chondrogenic differentiation of human bone-marrow-derived mesenchymal stem cells (hMSCs). The hMSCs were first cultured in collagen porous sponges and then embedded with or without collagen hydrogels for continual culture under different culture conditions. The different influences of collagen sponges, collagen hydrogels, and induction factors were investigated. The collagen sponges were beneficial for cell proliferation. The collagen sponges also promoted chondrogenic differentiation during culture in chondrogenic medium, which was superior to the effect of collagen sponges embedded with hydrogels without loading of induction factors. However, collagen sponges embedded with collagen hydrogels and loaded with induction factors had the same level of promotive effect on chondrogenic differentiation as collagen sponges during in vitro culture in chondrogenic medium and showed the highest promotive effect during in vivo subcutaneous implantation. The combination of collagen sponges with collagen hydrogels and induction factors could provide a platform for cell proliferation at an early stage and subsequent chondrogenic differentiation at a late stage. The results provide useful information for the chondrogenic differentiation of stem cells and cartilage tissue engineering.

## 1. Introduction

Cartilage tissue engineering using stem cells has been developed as an attractive approach for the treatment of cartilage defects because of the availability and pluripotency of stem cells [1,2,3]. Among the various types of stem cells, mesenchymal stem cells (MSCs) have the advantages of easy isolation from various tissues, a high capacity of self-renewal, a high potential of chondrogenic differentiation, and immunoregulatory and anti-inflammatory capacities [4,5]. MSCs can be isolated from bone marrow, adipose, synovium, infrapatellar fat pad, umbilical cord blood, etc. [6,7]. MSCs have been broadly studied as a useful cell source for cartilage repair and tissue engineering strategies [8,9].

Cell proliferation, chondrogenic differentiation, and maintenance of the differentiated phenotype of stem cells are critical for cartilage tissue engineering [10]. MSCs are generally cultured in three-dimensional scaffolds with chondrogenic induction (CI) factors and biomechanical stimuli to provide biomimetic conditions similar to those of in vivo cartilaginous microenvironments [11,12,13,14]. A variety of porous scaffolds and hydrogels have been reported for 3D culture of stem cells [15,16,17,18,19,20]. The scaffolds provide instructive biological and biomechanical signals to promote cell proliferation and trigger chondrogenic differentiation of stem cells.

Porous scaffolds for tissue engineering should have pore structures that facilitate cell infiltration and distribution throughout the scaffolds. The pore size is generally hundreds of micrometers. The pore structures provide spaces for accommodation of the proliferated cells and secreted extracellular matrices (ECM). In contrast, hydrogels are water-swollen networks that are covalently or physically crosslinked [21,22]. Cells embedded in hydrogels are surrounded by water-swollen networks that resemble the extracellular microenvironments surrounding cells in vivo [23,24]. The combination of porous scaffolds and hydrogels has been reported to facilitate cell loading [25,26]. In these studies, hydrogels have been used as a carrier to entrap the seeded cells in the porous structures, avoiding leakage of seeded cells from the interconnected pore structures of porous scaffolds.

Porous scaffolds and hydrogels have different effects on the proliferation and differentiation of stem cells due to their specific structural characteristics. Hydrogels not only promote cell encapsulation in porous scaffolds but also load induction factors for chondrogenic differentiation. Proliferation of stem cells requires more void spaces, while chondrogenic differentiation requires a 3D inductive microenvironment. The porous structures of porous scaffolds can provide sufficient void spaces for cell proliferation at an early stage of cell culture. After cell proliferation, chondrogenic differentiation is necessary at a late stage of cell culture and can be promoted by embedding in hydrogels with induction factors. Stepwise proliferation and chondrogenic differentiation can be realized by combining porous scaffolds and induction-factor-loaded hydrogels.

Therefore, in this study, collagen porous scaffolds (collagen sponges) and collagen hydrogels were combined to investigate their effect on the stepwise proliferation and chondrogenic differentiation of human bone-marrow-derived mesenchymal stem cells (hMSCs). Collagen was chosen because collagen is one of the predominant components of ECM including cartilaginous ECM. Collagen has high bioactivity and good biocompatibility. It has been used for the preparation of collagen-based porous scaffolds and hydrogels for tissue engineering [27,28,29,30,31,32]. The same collagen was used for the preparation of both porous scaffolds and hydrogels in this study to minimize the influence of different biomaterials. The hMSCs were first cultured in collagen sponges. Then, the cell-seeded collagen sponges after 4 weeks of culture were embedded with collagen hydrogels. The collagen hydrogels were loaded with or without (CI) factors to create different conditions for 3D culture of hMSCs. The influences of the different 3D culture conditions on the proliferation and chondrogenic differentiation of hMSCs were elucidated by investigating cell proliferation, cartilaginous matrix gene expression and secretion during in vitro culture and in vivo subcutaneous implantation.

## 2. Results and Discussion

### 2.1. Preparation and Characterization of Collagen Sponges

**Figure 1:**
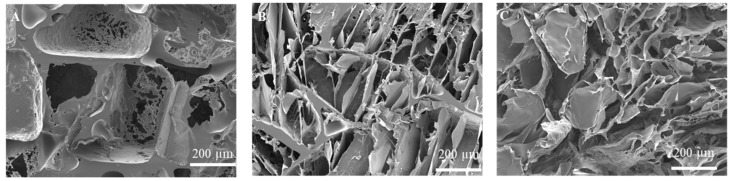
SEM images of cross-sections of (**A**) PLGA sponge, (**B**) PLGA-collagen sponge, and (**C**) collagen sponge.

Collagen sponges were prepared by using poly(lactic acid-*co*-glycolic acid) (PLGA) sponges as templates. PLGA sponges were prepared by a solvent casting/particulate leaching method using NaCl particulates. SEM (scanning electron microscope) observation showed the porous structure of the PLGA sponges (Figure 1A). Then, collagen sponge aqueous solution was introduced into the PLGA sponges, frozen, freeze-dried, and crosslinked to form PLGA-collagen sponges (Figure 1B). Finally, collagen sponges were generated after removal of PLGA sponge templates (Figure 1C). Removal of PLGA sponge templates left some interconnected channels in the collagen sponges. The interconnected pore structure in the collagen sponges could facilitate cell seeding and homogeneous cell distribution throughout the collagen sponges.

### 2.2. Cell Seeding and Distribution in Collagen Sponges

**Figure 2:**
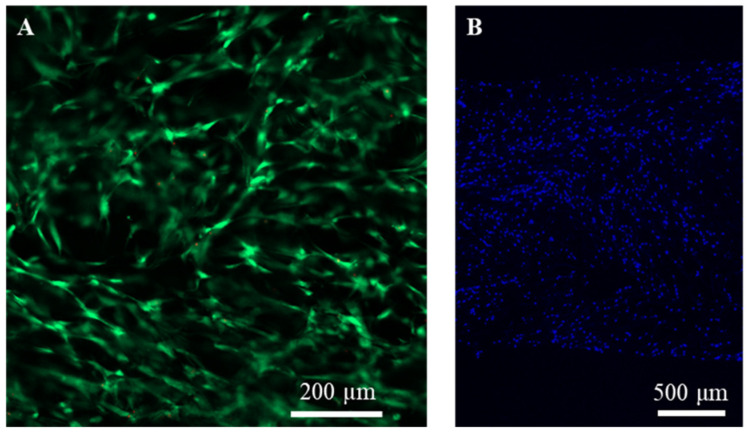
Fluorescence photomicrographs of (**A**) live/dead staining and (**B**) nuclear staining of hMSCs after culture in collagen sponges for 1 day. Green fluorescence indicates live cells, while red fluorescence indicates dead cells. Blue fluorescent dots indicate the stained nuclei.

The collagen sponges were cut into discs and used for the culture of hMSCs. The cells were seeded from both sides of collagen sponge discs. The cell seeding efficiency was 95.6 ± 1.3%. After 1 day of culture, cell viability and distribution in collagen sponges were investigated. No dead cells were detected. Almost all the cells were alive in the collagen sponges (Figure 2A). Nuclear staining showed homogeneous cell distribution from the upper surface to the bottom surface of the collagen sponges (Figure 2B). The high cell seeding efficiency and homogeneous cell distribution were due to the well interconnected porous structure of the collagen sponges. The interconnected pore structure allowed easy cell infiltration into the inner bulk pores throughout the scaffolds.

### 2.3. Gross Appearance, Cell Proliferation, ECM Secretion, and Mechanical Properties

**Figure 3:**
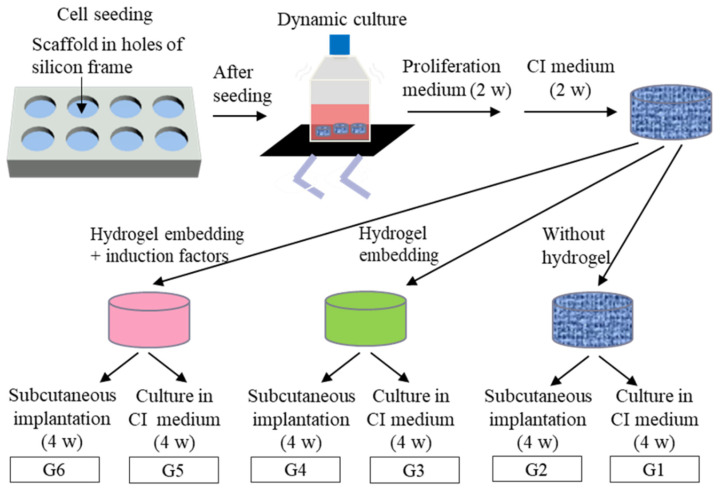
Scheme of in vitro cell culture and in vivo implantation of different samples.

**Figure 4:**
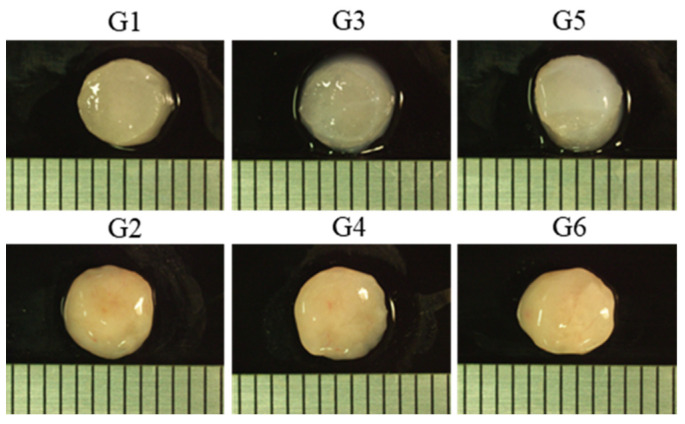
Gross appearances of the samples after in vitro culture for 8 weeks (G1, G3, and G5) and the samples after 4 weeks in vitro culture + 4 weeks in vivo subcutaneous implantation (G2, G4, and G6).

The hMSCs were cultured in collagen sponges in proliferation medium for 2 weeks and in CI medium for another 2 weeks. Then, the cell-seeded collagen sponges were continually cultured in CI medium for 4 weeks or embedded with collagen hydrogels with or without CI factors for continual culture or implantation. The samples of different conditions were divided into six groups. G1 and G2 were the cell-seeded collagen sponges without hydrogel for 4 weeks continual culture in CI medium or for 4 weeks implantation, respectively. G3 and G4 were the cell-seeded collagen sponges filled with hydrogel for 4 weeks continual culture in CI medium or for 4 weeks implantation, respectively. G5 and G6 were the cell-seeded collagen sponges filled with CI-factor-loaded hydrogel for 4 weeks continual culture in CI medium or for 4 weeks implantation, respectively. The detailed culture conditions of each group are shown in Figure 3. The cell/scaffold constructs after in vitro culture for 8 weeks (G1, 3 and 5 samples) and after in vivo subcutaneous implantation for 4 weeks (G2, 4 and 6 samples) showed a glistening white appearance (Figure 4). The in vivo implants had a more glistening appearance than the in vitro cultured constructs.

**Figure 5:**
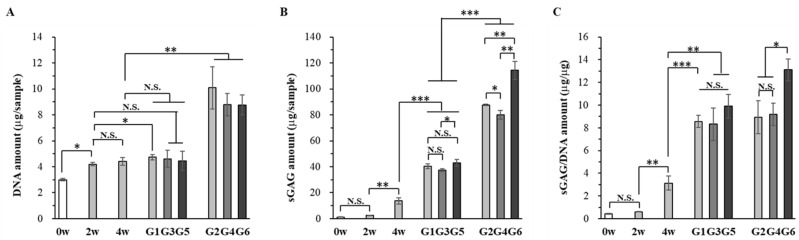
(**A**) DNA quantification, (**B**) sGAG amount quantification, and (**C**) sGAG/DNA ratio during in vitro culture for 8 weeks and in vivo subcutaneous implantation for 4 weeks. Data are shown as the mean ± SD (n = 3). Significant difference: *, *p* < 0.05; **, *p* < 0.01; ***, *p* < 0.001.

The DNA content and sulfated glycosaminoglycan (sGAG) amount in the cell/scaffold constructs after cell seeding (0 w sample), in vitro culture for 2, 4, and 8 weeks, and in vivo subcutaneous implantation for 4 weeks were measured (Figure 5). The DNA content increased significantly after the cells were cultured in the collagen sponge in proliferation medium for 2 weeks (2 w sample) (Figure 5A). Culture in CI medium for another 2 weeks (4 w sample) and 6 weeks without hydrogel (G1) slightly increased the DNA content. However, the DNA content in the collagen sponges embedded with hydrogel after 4 weeks of induction culture (G3 and G5 samples) was not significantly different from that of the 4 w sample, suggesting that the cells did proliferate during culture in the embedded hydrogels in the CI medium. After in vivo subcutaneous implantation, the DNA contents of the G2, G4, and G6 samples significantly increased. The G2 sample showed the highest DNA amount. The results indicated that hMSCs proliferated during in vitro culture in the collagen sponge in proliferation medium. Even during culture in CI medium, the cells in collagen sponges without hydrogels also slightly proliferated. However, the cells in the collagen sponges embedded with hydrogels almost did not proliferate during culture in CI medium. Implantation promoted cell proliferation, and the collagen sponge without hydrogel showed a higher promotive effect than the collagen sponge embedded with hydrogel with or without induction factors.

The amount of sGAG did not change significantly during the first 2 weeks of culture in proliferation medium but increased significantly during the 2 weeks of culture in CI medium (Figure 5B). Continual culture in CI medium for another 4 weeks without (G1 sample) or with hydrogel embedding (G3 and G5 samples) also significantly increased the sGAG amount. Subcutaneous implantation (G2, G4, and G6 samples) further significantly increased the sGAG amount. In particular, the G6 sample showed the highest sGAG amount. The results indicated that hMSCs produced less sGAG during in vitro culture in the collagen sponge in proliferation medium. The sGAG amount increased significantly during culture in induction medium with or without hydrogel. Implantation could further promote the secretion of sGAG. The sGAG amount in the collagen sponge embedded with hydrogel loaded with induction factors was the highest in the respective in vitro culture samples (G1, G3, and G5) or the in vivo implantation samples (G2, G4, and G6).

The value of sGAG/DNA reflects the sGAG secretion capacity of each cell. The sGAG/DNA ratio was calculated based on the DNA content and sGAG amount (Figure 5C). The sGAG/DNA ratio did not change significantly during in vitro 2 weeks culture in the proliferation medium. Culture in the induction medium significantly increased the secretion capacity of sGAG. During culture in CI medium or subcutaneous implantation, hMSCs cultured in the collagen sponges and hydrogel-embedded collagen sponges without loading of induction factors had the same capacity of sGAG secretion. However, loading of induction factors in the hydrogels further increased the sGAG secretion capacity. The implanted sample embedded with hydrogel and loaded with induction factors showed the highest capacity of sGAG secretion (G6 sample).

These results indicated that culture in collagen porous sponges in proliferation medium was beneficial for cell proliferation. Embedding in collagen hydrogel without induction factor loading did not promote sGAG secretion capacity during in vitro CI culture or in vivo subcutaneous implantation. Loading of induction factors in the hydrogels promoted sGAG secretion during in vitro CI culture and in vivo subcutaneous implantation. The results suggested that embedding of hydrogel plus loading of induction factors had the highest promotive effect on sGAG secretion.

**Figure 6:**
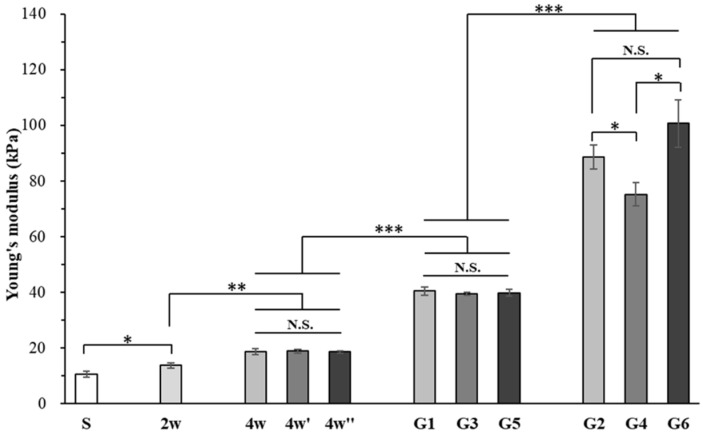
Young’s modulus of hydrated collagen sponges and cell/scaffold constructs after in vitro culture for 2, 4, and 8 weeks and in vivo subcutaneous implantation for 4 weeks. The 4 w’ and 4 w’’ samples are the 4 w samples immediately after embedding of induction-factor-free hydrogels and factor-loaded hydrogels, respectively. Data are shown as the mean ± SD (n = 3). Significant difference: *, *p* < 0.05; **, *p* < 0.01; ***, *p* < 0.001.

The Young’s modulus of in vitro cultured samples, implants, and hydrated collagen sponges without cells was measured by a compression test (Figure 6). Compared to the hydrated collagen sponge, the Young’s modulus of the cell/scaffold constructs after two weeks of culture in proliferation medium significantly increased (2 w sample). Culture in the CI medium further significantly increased the Young’s modulus (4 w sample). The Young’s modulus of the 4 w samples immediately after embedding of the hydrogel was also measured (samples 4 w’ and 4 w’’). Embedding of collagen hydrogel did not significantly affect the Young’s modulus of the cells/scaffold constructs (4 w, 4 w’, and 4 w’’ samples). Continual culture in CI medium for another 4 weeks and in vivo subcutaneous implantation significantly increased the Young’s modulus. Subcutaneously implanted samples showed the highest Young’s modulus. In particular, the G6 sample had the highest Young’s modulus among all the samples.

### 2.4. Gene Expression

**Figure 7:**
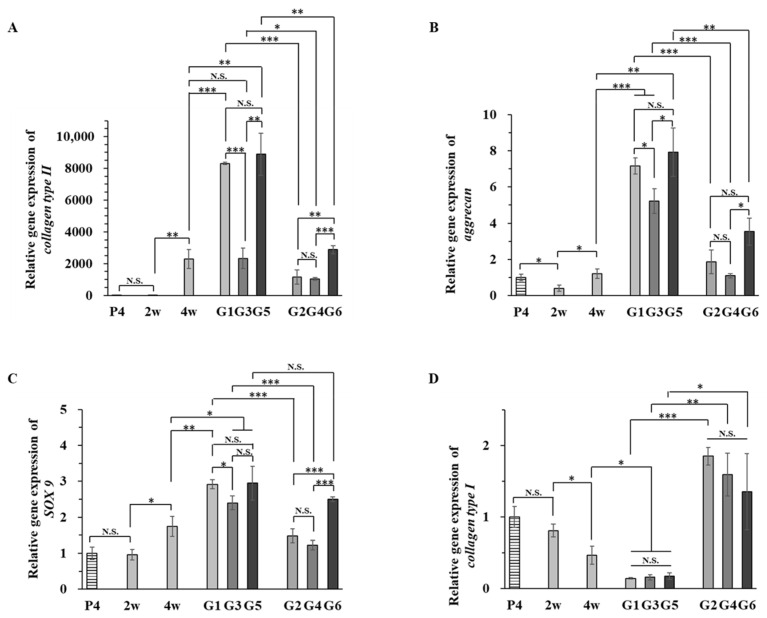
Gene expression of (**A**) collagen II, (**B**) aggrecan, (**C**) SOX9, and (**D**) collagen I of P4 hMSCs used for cell seeding and cell/scaffold constructs after in vitro culture for 2, 4, and 8 weeks and in vivo subcutaneous implantation for 4 weeks. Data are shown as the mean ± SD (n = 3). Significant difference: *, *p* < 0.05; **, *p* < 0.01; ***, *p* < 0.001.

The expression of the chondrogenic marker genes type II collagen, aggrecan, and SOX9 was analyzed by real-time PCR (Figure 7). The expression of the fibrous cartilage marker gene type I collagen was also investigated. The P4 hMSCs seeded in the scaffolds were used for comparison. When hMSCs were cultured in the collagen sponge in proliferation medium, the expression of collagen II, aggrecan, SOX9, and collagen I slightly decreased or did not change compared to that in P4 hMSCs, suggesting that culture in proliferation medium had no effect on chondrogenic differentiation. When the cells were cultured in CI medium, the expression of collagen II, aggrecan, and SOX9 increased, while that of type I collagen decreased, suggesting that culture in CI medium could effectively promote chondrogenic differentiation of hMSCs.

A long period of culture either without or with collagen hydrogels (G1, G3, and G5 samples) enhanced these effects. When the in vitro 8-week culture samples were compared, culture in collagen sponges without hydrogels (G1 sample) and culture in collagen sponges embedded with hydrogel and loaded with induction factors (G5 sample) resulted in higher expression levels of cartilaginous genes than culture in collagen sponge embedded with hydrogel without loading of induction factors (G3). After subcutaneous implantation, the expression of collagen II, aggrecan, and SOX9 was the same or slightly increased compared to that of the in vitro 4-week culture sample (4 w), while that of collagen I increased.

Compared to the in vitro 8-week culture samples (G1, G3, and G5), subcutaneous implantation decreased the expression of collagen II, aggrecan, and SOX9 and increased the expression of collagen I. When the G2, G4, and G6 samples were compared, the G2 and G6 samples had higher expression levels of cartilaginous genes than the G4 sample. The G6 sample showed the best promotive effect on the expression of these cartilaginous genes. Although the sGAG and sGAG/DNA ratios after subcutaneous implantation (G2, G4, and G6 samples) were much higher than those of in vitro cultured G1, G3, and G5 samples, the expression levels of cartilaginous genes were decreased after subcutaneous implantation. The DNA content also increased significantly after implantation. The changes might be due to immigration of surrounding cells during implantation.

### 2.5. Histological and Immunohistochemical Staining

**Figure 8:**
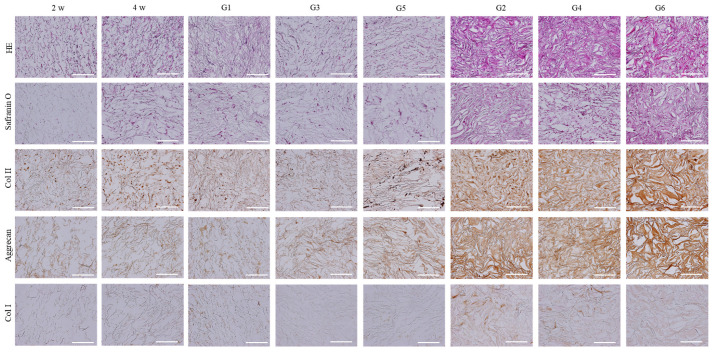
Optical photomicrographs of HE staining, safranin O staining, and immunohistochemical staining of collagen II, aggrecan, and collagen I in the cells/scaffold constructs after in vitro culture for 2, 4, and 8 weeks and in vivo subcutaneous implantation for 4 weeks. Scale bars = 200 µm.

HE staining showed the homogenous distribution of cells and ECM in the scaffolds. The subcutaneously implanted samples (G2, G4, and G6) showed denser ECM than the in vitro culture samples (G1, G3, and G5) (Figure 8). Safranin O staining and immunological staining of type II collagen and aggrecan showed the following trend: in vitro 2 week culture < in vitro 4 week culture < in vitro 8 week culture < subcutaneous implantation. Culture in a collagen sponge with a hydrogel having induction factors (G5 and G6 samples) showed the highest staining intensity in the in vitro 8-week culture and subcutaneous implantation samples (G1, G3, and G5; G2, G4, and G6). The immunological staining of collagen I showed that the subcutaneously implanted samples showed more intensive staining than the in vitro culture samples. Culture in collagen sponges embedded with hydrogels containing induction factors (G5 and G6 samples) showed the lowest staining intensity of collagen I in the in vitro 8-week culture and subcutaneous implantation samples.

Based on all these results, the effects of different culture conditions can be summarized as follows. First, culture in collagen porous sponges in proliferation medium was beneficial for proliferation. Second, culture in collagen sponges embedded with hydrogels containing induction factors had the highest promotive effect on chondrogenic differentiation. Third, culture in collagen sponges without hydrogel had the second highest promotive effect on chondrogenic differentiation. Fourth, culture in collagen sponges embedded with hydrogels without loading of induction factors had the lowest promotive effect on chondrogenic differentiation.

When hMSCs were cultured in collagen sponges, the interconnected porous structures of collagen sponges allowed the cells to infiltrate the scaffolds for homogenous distribution. The porous structure could also facilitate the diffusion of nutrients and removal of metabolic products, therefore promoting cell proliferation. Proliferation in porous scaffolds is important to obtain a sufficient cell number for cartilage tissue engineering. After an initial cell proliferation stage, the cells should be differentiated into the chondrogenic lineage, and their differentiated phenotype should be maintained. The different effects of the culture conditions on chondrogenic differentiation should be due to the respective characteristics of each condition. Porous scaffolds could allow easy diffusion of CI factors in the porous structure to access the cells. The loaded induction factors in the hydrogel could also be accessed by the cells. However, the porous scaffold embedded with hydrogel without induction factors could not directly provide induction factors to the cells. The induction factors needed to diffuse into the hydrogels for interaction with the cells. Compared with that of porous scaffolds, diffusion of induction factors from culture medium into hydrogels was slow.

The results suggested that both scaffolding conditions (porous scaffold or hydrogel) and supplementation with CI factors were important for chondrogenic differentiation. The cells in vivo are surrounded by ECM microenvironments that provide all necessary biological factors and biomechanical signals to maintain cell function and metabolism [33]. Scaffolds mimicking the natural ECM microenvironment can provide necessary signals to control cell functions and trigger chondrogenic differentiation. A variety of biological factors and physiochemical cues have been reported for their influences on the functions and differentiation of stem cells [34,35,36,37]. Supplementation with CI factors such as dexamethasone and TGF-β3 is important for chondrogenic differentiation of stem cells [38,39,40]. Induction factors are generally added to the culture medium during in vitro induction culture. Sequential provision of different induction factors in cycling has been shown to effectively stimulate the chondrogenic differentiation of hMSCs [41]. The collagen sponges embedded with induction-factor-loaded hydrogels could provide a more favorable chondrogenic inductive microenvironment for chondrogenic differentiation than other conditions in this study. Although embedding of collagen hydrogel had some promotive effect on chondrogenic differentiation, its effect was much weaker than that of CI factors.

Stem cell differentiation is a reversible process [42]. Not only the differentiation of stem cells but also the maintenance of their differentiated phenotypes is important for functional cartilage tissue engineering [43,44]. It has been reported that there may be difficulty in maintaining the differentiated or committed phenotype after the removal of biological induction factors [43]. Sustainable supplementation with induction factors is critical for a long period to maintain the differentiated phenotype [45,46]. However, after subcutaneous implantation, the concentrations of CI factors under physiological conditions are too low for continual stimulation of chondrogenic differentiation or maintenance of differentiated phenotypes [47,48]. In this study, compared with those of the in vitro cultured G1, G2, and G3 samples, the expression levels of cartilaginous genes after implantation decreased. This finding should be due to depletion of induction factors after implantation. CI factors have been introduced in porous scaffolds and hydrogels for local delivery of these factors to continually stimulate the implanted cells for effective chondrogenesis [9,49,50]. The highest promotive effect of the collagen sponges embedded with hydrogels loaded with induction factors could provide the sustainable delivery of induction factors after subcutaneous implantation to continually stimulate the implanted cells and maintain the differentiated phenotype. Compared to the 4 w sample, the cells cultured in the collagen sponges embedded with hydrogel loaded with induction factors had higher expression levels of cartilaginous genes, indicating that the phenotype of differentiated cells was maintained in the scaffolds.

## 3. Materials and Methods

### 3.1. Scaffold Preparation

Interconnected collagen porous scaffolds were prepared by a template method [51]. First, porous sponge templates of PLGA were used with a solvent casting/particulate leaching method. PLGA (1 g) was dissolved in chloroform (4.5 mL) and mixed with NaCl particles (9.0 g) with a size of 355-500 μm. The mixture was cast in an aluminum pan and dried in air. NaCl particles were leached by washing with water to form PLGA sponges. Subsequently, the PLGA sponges were filled with collagen solution (1 (wt/v) %, Nippi, Tokyo, Japan) under vacuum. The collagen-solution-filled PLGA sponges were frozen at −80 °C and freeze-dried to form collagen sponges in the porous spaces of PLGA sponges (PLGA-collagen sponge). After freeze-drying, the PLGA-collagen sponges were crosslinked by immersion in a series of ethanol aqueous solutions at concentrations of 95, 90, and 80 (*v*/*v*)% containing 50 mM 1-ethyl-3-(3-dimethylaminopropyl) carbodiimide (EDC, Peptide Institute, Osaka, Japan) and 20 mM N-hydroxysuccinimide (NHS, FUJIFILM Wako Pure Chemical, Osaka, Japan). The crosslinking time in each solution was 3 h. After crosslinking, the PLGA-collagen sponges were immersed in 0.1 M glycine aqueous solution for 12 h to block the activated groups. Finally, the PLGA sponge templates were removed by immersion in ammonia hydroxide aqueous solution (3 (wt/v)%) for 2 days under shaking. Collagen sponges with an interconnected pore structure were formed after the removal of PLGA templates from the PLGA-collagen sponges. The collagen sponges were washed with water 6 times, freeze-dried, and stored at 4 °C for the following experiments. The pore structures of the PLGA templates, PLGA-collagen sponges, and collagen sponges were observed by scanning electron microscopy (JSM-6400Fs, JEOL, Tokyo, Japan).

### 3.2. In Vitro Culture of hMSCs in Collagen Sponges

The collagen sponges were cut into cylindrical discs with a diameter of 6 mm and a thickness of 3 mm. The collagen sponge discs were sterilized with 70% ethanol aqueous solution and used for the culture of hMSCs. The collagen sponge discs were placed in the holes of silicone frames that were used to protect the leakage of the cell suspension solution from the sponge discs (Figure 3). The silicone holes had a diameter of 6 mm and a height of 4 mm. The hMSCs (Lonza, Walkersville, MD, USA) were subcultured in MSCGM^TM^ culture medium (Lonza, Walkersville, MD, USA) in a CO_2_ incubator with 5% CO_2_ at 37 °C. The subcultured P4 hMSCs were harvested and suspended in the culture medium at a concentration of 5 × 10^6^ cells mL^−1^. The hMSCs were seeded in collagen sponge discs by adding 100 μL of cell suspension solution on each side of the scaffold discs (1 × 10^6^ cells/scaffold). After each time of cell seeding, the cells were cultured for 6 h. Then, the cell-seeded scaffolds were transferred from the silicone frames to cell culture flasks and cultured in serum-containing DMEM (10% fetal bovine serum, proliferation medium). The culture was continued under shaking at a speed of 60 rpm for 2 weeks. The culture medium was refreshed every 3 days.

To measure cell seeding efficiency, the cells that did not attach to the scaffolds were collected from the silicone frames and counted (unattached cell number). The attached cell number was calculated by subtracting the unattached cell number from the total number of seeded cells. Cell seeding efficiency was calculated by dividing the number of attached cells by the total number of seeded cells. Cell viability after 1 day of culture was evaluated by live/dead staining. After 1 day of culture, the cell/scaffold constructs were washed three times with PBS and cultured with serum-free DMEM containing calcein-AM and propidium iodide at 37 °C for 15 min. After PBS washes, the stained cells in the scaffolds were observed with a fluorescence microscope. Cell distribution in the scaffolds after 1 day of culture was examined by nuclear staining. After 1 day of culture, the cells/scaffold constructs were washed three times with PBS, and the cells were fixed with neutral buffered formalin (10%) at room temperature for 24 h. After fixation, the cell/scaffold constructs were embedded in paraffin, cut into 7-μm-thick slices, deparaffinized, and stained with 4′,6-diamidino-2-phenylindole dihydrochloride (DAPI, 1 μg mL^−1^, 10 m). The stained slices were observed with a fluorescence microscope.

After 2 weeks of culture, the culture medium was changed to CI medium. The CI medium was prepared by supplementing high glucose DMEM (serum-free) with 4 mM L-glutamine, 100 U mL^−1^ penicillin, 100 µg mL^−1^ streptomycin, 0.4 mM proline, 0.1 mM nonessential amino acids, 50 mg mL^−1^ ascorbic acid, 10^−7^ M dexamethasone, 10 ng mL^−1^ TGF-β3, and 1% ITS (Lonza).

### 3.3. In Vitro Culture in Collagen Hydrogels

After the cell-seeded scaffolds were cultured in proliferation medium for 2 weeks and CI medium for another 2 weeks, the cells/scaffold constructs were divided into six groups. The detailed culture conditions of each group are shown in Figure 3. The first group was the cell-seeded collagen sponges without hydrogel introduction, which were continually cultured in CI medium under shaking for 4 weeks (G1). The second group was the cell-seeded collagen sponges without hydrogel introduction, which were subcutaneously implanted in the backs of nude mice for 4 weeks (G2). The third group was the cell-seeded collagen sponges that were filled with collagen hydrogel without CI factors and continually cultured in CI medium under shaking for 4 weeks (G3). The fourth group was the cell-seeded collagen sponges that were filled with collagen hydrogel without CI factors and subcutaneously implanted in the backs of nude mice for 4 weeks (G4). The fifth group was the cell-seeded collagen sponges that were filled with collagen hydrogel loaded with CI factors and continually cultured in CI medium under shaking for 4 weeks (G5). The six groups were the cell-seeded collagen sponges that were filled with collagen hydrogel loaded with CI factors and subcutaneously implanted in the backs of nude mice for 4 weeks (G6).

Before the introduction of collagen hydrogel in the cell-seeded collagen sponges, the medium in the cell/scaffold constructs was aspirated with sterile filter paper. Then, the cell-seeded collagen sponge discs were placed in the abovementioned silicone frames, and the hydrogel-forming solution (80 μL/scaffold) was added. The hydrogel-forming solution was prepared by mixing cold collagen solution (0.5 (wt/v)%) with 10X concentrated serum-free DMEM and 10X concentrated PBS on ice. After the hydrogel-forming solution penetrated the cells/scaffold constructs, the constructs were incubated in a CO_2_ incubator at 37 °C for 2 h to allow the formation of collagen hydrogel in the cells/scaffold constructs. The collagen hydrogel loaded with CI factors was introduced into the cell-seeded collagen sponge discs as described above by using hydrogel-forming solution containing CI factors that was prepared by mixing cold collagen solution (0.5 (wt/v)%) with 10X concentrated CI DMEM and 10X concentrated PBS on ice.

### 3.4. Animal Implantation Experiment

Animal experiments were approved by the animal experiment ethical committee of the National Institute for Materials Science and conducted under the institute guidelines. The above-described G2, 4, and 6 samples were subcutaneously implanted on the backs of nude mice that were purchased from Charles River Laboratories (Yokohama, Japan). Each mouse was implanted with three samples (each sample from G2, 4, and 6). After 4 weeks of implantation, the samples were harvested, and their gross appearance was examined by optical microscopy.

### 3.5. Measurement of DNA, Sulfated Glycosaminoglycan (sGAG) Amount, and Mechanical Strength

The cell/scaffold constructs after cell seeding (0 w sample), in vitro culture for 2 (2 w sample), 4 (4 w sample), and 8 weeks and in vivo subcutaneous implantation for 4 weeks were harvested for quantification of DNA and sGAG amounts. The harvested cell/scaffold constructs were washed, freeze-dried, and digested with papain solution at 60 °C under shaking for 6 h. The papain solution was prepared by dissolving papain in 0.1 M PBS buffer (400 µg/mL) with L-cysteine hydrochloride monohydrate (5 mM) and EDTA (5 mM) at a pH of 6. The amount of DNA and sGAG in the digestion solution was quantified with Hoechst 33,258 and a Blyscan^TM^ Glycosaminoglycan Assay Kit, respectively.

The cell/scaffold constructs after in vitro culture for 2, 4, and 8 weeks and in vivo subcutaneous implantation for 4 weeks were washed thrice with PBS and compression testing was applied. The collagen scaffold discs without cell seeding were hydrated in PBS and used for the mechanical testing as a control (S sample). The samples were compressed at a rate of 0.1 mm/s using a texture analyzer (TA. XTPlus, Texture Technologies, Hamilton, MA, USA). Young’s modulus was calculated from the initial linear region of the stress–strain curves. Triplicate samples were used for each of these measurements to calculate means and standard deviations.

### 3.6. Analysis of Gene Expression by Real-Time PCR

The cell/scaffold constructs after in vitro culture for 2, 4, and 8 weeks and in vivo subcutaneous implantation for 4 weeks were harvested for analysis of gene expression. The P4 hMSCs used for cell seeding were used as a control. The samples were washed three times with PBS and frozen in liquid nitrogen. The frozen samples were pulverized with an electric crusher, and Sepasol-RNA I Super G solution (1 mL) was added. Total RNA was extracted according to a reported protocol [14]. After reverse transcription with a high-capacity cDNA reverse transcription kit, amplification of glyceraldehyde-3-phosphate dehydrogenase (GAPDH, a housekeeping gene), collagen I (Col1a2), collagen II (Col2a1), aggrecan (Acan), and SOX9 was conducted with a QuantStudio^®^ 3 Real-Time PCR System (Thermo Fisher Scientific). The previously reported primer and probe sequences as shown below were used [52,53]. GAPDH: (forward) 5′-ATGGGGAAGGTGAAGGTCG-3′, (reverse) 5′-TAAAAGCAGCCCTGGTGACC-3′, (probe) 5′-CGCCCAATACGACCAAATCCGTTGAC-3′; Col1a2: (forward) 5′-CAGCCGCTTCACCTACAGC-3′, (reverse): 5′-TTTTGTATTCAATCACTGTCTTGCC-3′, (probe): 5′−CCGGTGTGACTCGTGCAGCCATC-3′; Col2a1: (forward) 5′GGCAATAGCAGGTTCACGTACA-3′, (reverse) 5′-CGATAACAGTCTTGCCCCACTT-3′, (probe) 5′−CCGGTATGTTTCGTGCAGCCATCCT-3′; Acan: (forward) 5′-TCGAGGACAGCGAGGCC-3′, (reverse) 5′-TCGAGGGTGTAGCGTGTAGAGA-3′, (probe) 5′-ATGGAACACGATGCCTTTCACCACGA-3′; SOX9: (forward) 5′-CACACAGCTCACTCGACCTTG-3′, (reverse) 5′-TTCGGTTATTTTTAGGATCATCTCG-3′, (probe) 5′-CCCACGAAGGGCGACGATGG-3′. A 2^−∆∆Ct^ method was used to calculate the relative expression of each gene with an endogenous control (GAPDH). The expression level of each gene was normalized by using the respective gene expression of the P4 hMSCs as a comparison. Triplicate samples were used to calculate the means and standard deviations.

### 3.7. Histological and Immunohistochemical Staining

The cell/scaffold constructs after in vitro culture for 8 weeks and in vivo subcutaneous implantation for 4 weeks were applied for histological and immunohistochemical staining. The samples were washed three times with PBS and fixed with 10% neutral buffered formalin at room temperature for 24 h. The fixed samples were dehydrated, embedded in paraffin, and cut into 7-μm-thick slices. The sliced cross-sections were deparaffinized and stained with hematoxylin/eosin and safranin O/light green.

Collagen II, aggrecan, and collagen I were immunohistologically stained with anti-collagen II, anti-aggrecan, and anti-collagen I antibodies, respectively. First, the sliced cross-sections were treated with proteinase K (Dako, Carpinteria, CA, USA) for 5 min for antigen retrieval, followed by incubation in 1% BSA/PBS at room temperature for 1 h for blocking. Then, the cross-sections were immersed in 0.1% BSA/PBS containing the primary antibodies at room temperature for 2 h. Primary antibodies against collagen II (Invitrogen, Carlsbad, CA, USA), aggrecan (Abcam, Cambridge, UK), and collagen I (Abcam, Cambridge, UK) were used. After PBS washes, the cross-sections were immersed in peroxidase-conjugated secondary antibody (Dako, Carpinteria, CA, USA) at room temperature for 1 h. Finally, the cross-sections were immersed in 3,3′-diaminobenzidine substrate-chromogen for color development. The stained slices were mounted with Vectashield mounting medium (Vector, Burlingame, CA, USA) and observed with an optical microscope.

### 3.8. Statistics

The quantitative data were statistically analyzed by one-way analysis of variance (ANOVA) using KyPlot 5.0 (KyensLab, Inc., Tokyo, Japan). The data are shown as the means ± standard deviations (S.D.), and significant differences are expressed as * (*p* < 0.05), ** (*p* < 0.01), and *** (*p* < 0.001).

## 4. Conclusions

In summary, the influences of collagen sponges, collagen sponges embedded with collagen hydrogels, and collagen sponges embedded with collagen hydrogels and loaded with CI factors on the proliferation and chondrogenic differentiation of hMSCs were investigated during in vitro cell culture and in vivo subcutaneous implantation. Collagen sponges were beneficial for the proliferation of hMSCs at an early stage of cell culture. Collagen sponges embedded with induction-factor-loaded hydrogels facilitated chondrogenic differentiation under in vitro cell culture and in vivo subcutaneous implantation at a late stage. Stepwise proliferation and chondrogenic differentiation were realized by combining collagen sponges and collagen hydrogels with induction factors. Collagen sponges embedded with induction-factor-loaded hydrogels should be a useful platform for chondrogenic differentiation of stem cells and cartilage tissue engineering.

## Data Availability

The data presented in this study are available on request from the corresponding author. The data are not publicly available due to its confidentiality.

## References

[1] Canadas R.F., Pirraco R.P., Oliveira J.M., Reis R.L., Marques A.P., Oliveira J.M., Pina S., Reis R.L., Roman J.S. (2018). Stem Cells for Osteochondral Regeneration. Osteochondral Tissue Engineering: Challenges, Current Strategies, and Technological Advances.

[2] Kuroda R., Ishida K., Matsumoto T., Akisue T., Fujioka H., Mizuno K., Ohgushi H., Wakitani S., Kurosaka M. (2007). Treatment of a full-thickness articular cartilage defect in the femoral condyle of an athlete with autologous bone-marrow stromal cells. Osteoarthr. Cartil..

[3] Wakitani S., Mitsuoka T., Nakamura N., Toritsuka Y., Nakamura Y., Horibe S. (2004). Autologous bone marrow stromal cell transplantation for repair of full-thickness articular cartilage defects in human patellae: Two case reports. Cell Transplant..

[4] Ding N., Li E.M., Ouyang X.B., Guo J., Wei B. (2021). The Therapeutic Potential of Bone Marrow Mesenchymal Stem Cells for Articular Cartilage Regeneration in Osteoarthritis. Curr. Stem Cell Res. Ther..

[5] Aldrich E.D., Cui X.L., Murphy C.A., Lim K.S., Hooper G.J., McIlwraith C.W., Woodfield T.B.F. (2021). Allogeneic mesenchymal stromal cells for cartilage regeneration: A review of in vitro evaluation, clinical experience, and translational opportunities. Stem Cells Transl. Med..

[6] Urlic I., Ivkovic A. (2021). Cell Sources for Cartilage Repair-Biological and Clinical Perspective. Cells.

[7] Vahedi P., Moghaddamshahabi R., Webster T.J., Koyuncu A.C.C., Ahmadian E., Khan W.S., Mohamed A.J., Eftekhari A. (2021). The Use of Infrapatellar Fat Pad-Derived Mesenchymal Stem Cells in Articular Cartilage Regeneration: A Review. Int. J. Mol. Sci..

[8] Zha K.K., Sun Z.Q., Yang Y., Chen M.X., Gao C.J., Fu L.W., Li H., Sui X., Guo Q.Y., Liu S.Y. (2021). Recent Developed Strategies for Enhancing Chondrogenic Differentiation of MSC: Impact on MSC-Based Therapy for Cartilage Regeneration. Stem Cells Int..

[9] Kim Y.S., Mikos A.G. (2021). Emerging strategies in reprogramming and enhancing the fate of mesenchymal stem cells for bone and cartilage tissue engineering. J. Control. Release.

[10] Madry H., Rey-Rico A., Venkatesan J.K., Johnstone B., Cucchiarini M. (2014). Transforming Growth Factor Beta- Releasing Scaffolds for Cartilage Tissue Engineering. Tissue Eng. Part B-Rev..

[11] Lee K., Chen Y.Z., Li X.M., Kawazoe N., Yang Y.N., Chen G.P. (2021). Influence of viscosity on chondrogenic differentiation of mesenchymal stem cells during 3D culture in viscous gelatin C solution-embedded hydrogels. J. Mater. Sci. Technol..

[12] Huang K., Li Q., Li Y., Yao Z.H., Luo D.W., Rao P.C., Xiao J.G. (2018). Cartilage Tissue Regeneration: The Roles of Cells, Stimulating Factors and Scaffolds. Curr. Stem Cell Res. Ther..

[13] Li X.M., Zhang J., Kawazoe N., Chen G.P. (2017). Fabrication of Highly Crosslinked Gelatin Hydrogel and Its Influence on Chondrocyte Proliferation and Phenotype. Polymers.

[14] Makris E.A., Gomoll A.H., Malizos K.N., Hu J.C., Athanasiou K.A. (2015). Repair and tissue engineering techniques for articular cartilage. Nat. Rev. Rheumatol..

[15] Intini C., Lemoine M., Hodgkinson T., Casey S., Gleeson J.P., O’Brien F.J. (2022). A highly porous type II collagen containing scaffold for the treatment of cartilage defects enhances MSC chondrogenesis and early cartilaginous matrix deposition. Biomater. Sci..

[16] Shestovskaya M.V., Bozhkova S.A., Sopova J.V., Khotin M.G., Bozhokin M.S. (2021). Methods of Modification of Mesenchymal Stem Cells and Conditions of Their Culturing for Hyaline Cartilage Tissue Engineering. Biomedicines.

[17] Teng B.H., Zhang S.Q., Pan J.J., Zeng Z.Q., Chen Y., Hei Y., Fu X.M., Li Q., Ma M., Sui Y. (2021). A chondrogenesis induction system base d on a functionalize d hyaluronic acid hydrogel sequentially promoting hMSC proliferation, condensation, differentiation, and matrix deposition. Acta Biomater..

[18] Chen G.P., Kawazoe N., Oliveira J.M., Pina S., Reis R.L., SanRoman J. (2018). Porous Scaffolds for Regeneration of Cartilage, Bone and Osteochondral Tissue. Osteochondral Tissue Engineering: Nanotechnology, Scaffolding-Related Developments and Translation.

[19] Cai R., Nakamoto T., Kawazoe N., Chen G.P. (2015). Influence of stepwise chondrogenesis-mimicking 3D extracellular matrix on chondrogenic differentiation of mesenchymal stem cells. Biomaterials.

[20] Kawazoe N., Inoue C., Tateishi T., Chen G.P. (2010). A Cell Leakproof PLGA-Collagen Hybrid Scaffold for Cartilage Tissue Engineering. Biotechnol. Prog..

[21] Huang J.H., Xiong J.Y., Wang D.P., Zhang J., Yang L., Sun S.Q., Liang Y.J. (2021). 3D Bioprinting of Hydrogels for Cartilage Tissue Engineering. Gels.

[22] Bao W.R., Li M.L., Yang Y.Y., Wan Y., Wang X., Bi N., Li C.L. (2020). Advancements and Frontiers in the High Performance of Natural Hydrogels for Cartilage Tissue Engineering. Front. Chem..

[23] Yang J.Z., Zhang Y.S., Yue K., Khademhosseini A. (2017). Cell-laden hydrogels for osteochondral and cartilage tissue engineering. Acta Biomater..

[24] Shi W., Fang F., Kong Y.F., Greer S.E., Kuss M., Liu B., Xue W., Jiang X.P., Lovell P., Mohs A.M. (2022). Dynamic hyaluronic acid hydrogel with covalent linked gelatin as an anti-oxidative bioink for cartilage tissue engineering. Biofabrication.

[25] Gossla E., Bernhardt A., Tonndorf R., Aibibu D., Cherif C., Gelinsky M. (2021). Anisotropic Chitosan Scaffolds Generated by Electrostatic Flocking Combined with Alginate Hydrogel Support Chondrogenic Differentiation. Int. J. Mol. Sci..

[26] Moutos F.T., Freed L.E., Guilak F. (2007). A biomimetic three-dimensional woven composite scaffold for functional tissue engineering of cartilage. Nat. Mater..

[27] Xie Y., Kawazoe N., Yang Y.N., Chen G.P. (2022). Preparation of mesh-like collagen scaffolds for tissue engineering. Mater. Adv..

[28] Lin K.L., Zhang D.W., Macedo M.H., Cui W.G., Sarmento B., Shen G.F. (2019). Advanced Collagen-Based Biomaterials for Regenerative Biomedicine. Adv. Funct. Mater..

[29] Lausch A.J., Chong L.C., Uludag H., Sone E.D. (2018). Multiphasic Collagen Scaffolds for Engineered Tissue Interfaces. Adv. Funct. Mater..

[30] Dong C.J., Lv Y.G. (2016). Application of Collagen Scaffold in Tissue Engineering: Recent Advances and New Perspectives. Polymers.

[31] Sato T., Chen G.P., Ushida T., Ishii T., Ochiai N., Tateishi T., Tanaka J. (2004). Evaluation of PLLA-collagen hybrid sponge as a scaffold for cartilage tissue engineering. Mater. Sci. Eng. C.

[32] Lu H.X., Ko Y.G., Kawazoe N., Chen G.P. (2010). Cartilage tissue engineering using funnel-like collagen sponges prepared with embossing ice particulate templates. Biomaterials.

[33] Redini F., Daireaux M., Mauviel A., Galera P., Loyau G., Pujol J.P. (1991). Characterization of proteoglycans synthesized by rabbit articular chondrocytes in response to transforming growth-factor-beta (TGF-BETA). Biochim. Biophys. Acta.

[34] Higuchi A., Ling Q.D., Kumar S.S., Chang Y., Alarfaj A.A., Munusamy M.A., Murugan K., Hsu S.T., Umezawa A. (2015). Physical cues of cell culture materials lead the direction of differentiation lineages of pluripotent stem cells. J. Mat. Chem. B.

[35] Higuchi A., Ling Q.D., Chang Y., Hsu S.T., Umezawa A. (2013). Physical Cues of Biomaterials Guide Stem Cell Differentiation Fate. Chem. Rev..

[36] Higuchi A., Ling Q.D., Hsu S.T., Umezawa A. (2012). Biomimetic Cell Culture Proteins as Extracellular Matrices for Stem Cell Differentiation. Chem. Rev..

[37] Guo L.K., Kawazoe N., Fan Y.J., Ito Y., Tanaka J., Tateishi T., Zhang X.D., Chen G.P. (2008). Chondrogenic differentiation of human mesenchymal stem cells on photoreactive polymer-modified surfaces. Biomaterials.

[38] Uzieliene I., Bironaite D., Bernotas P., Sobolev A., Bernotiene E. (2021). Mechanotransducive Biomimetic Systems for Chondrogenic Differentiation In Vitro. Int. J. Mol. Sci..

[39] Calabrese G., Forte S., Gulino R., Cefali F., Figallo E., Salvatorelli L., Maniscalchi E.T., Angelico G., Parenti R., Gulisano M. (2017). Combination of Collagen-Based Scaffold and Bioactive Factors Induces Adipose-Derived Mesenchymal Stem Cells Chondrogenic Differentiation In vitro. Front. Physiol..

[40] Wang C.H., Tsai C.H., Lin T.L., Liu S.P. (2021). The Effects of Tgfb1 and Csf3 on Chondrogenic Differentiation of iPS Cells in 2D and 3D Culture Environment. Int. J. Mol. Sci..

[41] Indrawattana N., Chen G.P., Tadokoro M., Shann L.H., Ohgushi H., Tateishi T., Tanaka J., Bunyaratvej A. (2004). Growth factor combination for chondrogenic induction from human mesenchymal stem cell. Biochem. Biophys. Res. Commun..

[42] Tagami M., Ichinose S., Yamagata K., Fujino H., Shoji S., Hiraoka M., Kawano S. (2003). Genetic and ultrastructural demonstration of strong reversibility in human mesenchymal stem cell. Cell Tissue Res..

[43] Murphy K.C., Hoch A.I., Harvestine J.N., Zhou D.J., Leach J.K. (2016). Mesenchymal Stem Cell Spheroids Retain Osteogenic Phenotype Through alpha(2)beta(1) Signaling. Stem Cells Transl. Med..

[44] Yang Y.J., Wang X.L., Wang Y.T., Hu X.H., Kawazoe N., Yang Y.N., Chen G.P. (2019). Influence of Cell Spreading Area on the Osteogenic Commitment and Phenotype Maintenance of Mesenchymal Stem Cells. Sci. Rep..

[45] Liang M.S., Andreadis S.T. (2011). Engineering fibrin-binding TGF-beta 1 for sustained signaling and contractile function of MSC based vascular constructs. Biomaterials.

[46] Askari M., Bonakdar S., Anbouhi M.H., Shahsavarani H., Kargozar S., Khalaj V., Shokrgozar M.A. (2019). Sustained release of TGF-1 via genetically-modified cells induces the chondrogenic differentiation of mesenchymal stem cells encapsulated in alginate sulfate hydrogels. J. Mater. Sci.-Mater. Med..

[47] Tang X.D., Muhammad H., McLean C., Miotla-Zarebska J., Fleming J., Didangelos A., Onnerfjord P., Leask A., Saklatvala J., Vincent T.L. (2018). Connective tissue growth factor contributes to joint homeostasis and osteoarthritis severity by controlling the matrix sequestration and activation o latent TGF beta. Ann. Rheum. Dis..

[48] Wu M.R., Chen G.Q., Li Y.P. (2016). TGF-beta and BMP signaling in osteoblast, skeletal development, and bone formation, homeostasis and disease. Bone Res..

[49] Hauptstein J., Forster L., Nadernezhad A., Groll J., Tessmar J., Blunk T. (2022). Tethered TGF-beta 1 in a Hyaluronic Acid-Based Bioink for Bioprinting Cartilaginous Tissues. Int. J. Mol. Sci..

[50] Jung H., McClellan P., Welter J.F., Akkus O. (2021). Chondrogenesis of Mesenchymal Stem Cells through Local Release of TGF-beta 3 from Heparinized Collagen Biofabric. Tissue Eng. Part A.

[51] Xie Y., Lee K.B., Wang X.H., Yoshitomi T., Kawazoe N., Yang Y.N., Chen G.P. (2021). Interconnected collagen porous scaffolds prepared with sacrificial PLGA sponge templates for cartilage tissue engineering. J. Mat. Chem. B.

[52] Xie Y., Sutrisno L., Yoshitomi T., Kawazoe N., Yang Y.N., Chen G.P. (2022). Three-dimensional culture and chondrogenic differentiation of mesenchymal stem cells in interconnected collagen scaffolds. Biomed. Mater..

[53] Lu H.X., Hoshiba T., Kawazoe N., Koda I., Song M.H., Chen G.P. (2011). Cultured cell-derived extracellular matrix scaffolds for tissue engineering. Biomaterials.

